# Percentage-based Author Contribution Index: a universal measure of author contribution to scientific articles

**DOI:** 10.1186/s41073-017-0042-y

**Published:** 2017-11-03

**Authors:** Stéphane Boyer, Takayoshi Ikeda, Marie-Caroline Lefort, Jagoba Malumbres-Olarte, Jason M. Schmidt

**Affiliations:** 1 Institut de Recherche sur la Biologie de l’Insecte (IRBI) – UMR 7261 CNRS / Université de Tours, Parc Grandmont, 37200 Tours, France; 2 Applied Molecular Solutions Research Group, Environmental and Animal Sciences, Unitec Institute of Technology, Private Bag 92025, Victoria Street West, Auckland, 1142 New Zealand; 3 Japan Agency for Marine-Earth Science and Technology, Yokohama Institute for Earth Sciences, 3173-25 Showa-machi, Kanazawa-ku, Yokohama, 236-0001 Japan; 4 Bio-Protection Research Centre, Lincoln University, P. O. Box 85084, Christchurch, 7647 New Zealand; 5 Department of Evolutionary Biology, Ecology and Environmental Sciences, and Biodiversity Research Institute (IRBio), University of Barcelona, Avd. Diagonal, 643, 08028 Barcelona, Spain; 6 Center for Macroecology, Evolution and Climate, Natural History Museum of Denmark, University of Copenhagen, Copenhagen, Denmark; 7 Department of Entomology, The University of Georgia, Coastal Plain Experimental Station, 2360 Rainwater Rd, Tifton, GA 31793 USA

**Keywords:** Co-authorship, Author contribution, Publication metric, Scientific integrity

## Abstract

**Background:**

Deciphering the amount of work provided by different co-authors of a scientific paper has been a recurrent problem in science. Despite the myriad of metrics available, the scientific community still largely relies on the position in the list of authors to evaluate contributions, a metric that attributes subjective and unfounded credit to co-authors. We propose an easy to apply, universally comparable and fair metric to measure and report co-authors contribution in the scientific literature.

**Methods:**

The proposed Author Contribution Index (ACI) is based on contribution percentages provided by the authors, preferably at the time of submission. Researchers can use ACI to compare the contributions of different authors, describe the contribution profile of a particular researcher or analyse how contribution changes through time. We provide such an analysis based on contribution percentages provided by 97 scientists from the field of ecology who voluntarily responded to an online anonymous survey.

**Results:**

ACI is simple to understand and to implement because it is based solely on percentage contributions and the number of co-authors. It provides a continuous score that reflects the contribution of one author as compared to the average contribution of all other authors. For example, ACI(i) = 3, means that author i contributed three times more than what the other authors contributed on average. Our analysis comprised 836 papers published in 2014-2016 and revealed patterns of ACI values that relate to career advancement.

**Conclusion:**

There are many examples of author contribution indices that have been proposed but none has really been adopted by scientific journals. Many of the proposed solutions are either too complicated, not accurate enough or not comparable across articles, authors and disciplines. The author contribution index presented here addresses these three major issues and has the potential to contribute to more transparency in the science literature. If adopted by scientific journals, it could provide job seekers, recruiters and evaluating bodies with a tool to gather information that is essential to them and cannot be easily and accurately obtained otherwise. We also suggest that scientists use the index regardless of whether it is implemented by journals or not.

**Electronic supplementary material:**

The online version of this article (10.1186/s41073-017-0042-y) contains supplementary material, which is available to authorized users.

## Background

Deciphering the role and quantifying the amount of work provided by different co-authors of a particular paper has been a recurrent problem for the scientific community [1–3]. The position in the list of authors is commonly used to infer co-authors’ contribution and a number of systems have been proposed on this basis. They range from simple calculations based on the rank of the authors such as harmonic authorship credit, fractional authorship credit and inflated authorship [4] to more complex credits (e.g. [5]), some even taking into account the controversial journal impact factor [2]. However, these metrics are essentially ‘one fits all’ approaches that assume the contribution of each author based on their position in the author list and attributes subjective and unfounded values to these positions. As such, they do not attempt to represent and quantify ‘true’ contribution. Despite the growing interest in resolving the issue of authorship contributions in scientific disciplines [3, 4, 6], no standard ranking system has been widely recognised or adopted by scientific journals. With this lack of consensus, some journals have implemented a compulsory or recommended section dedicated to reporting authors’ contribution.

A review of the top 150 ecology journals referenced in ISI Web Of Knowledge revealed that 13.3% of them require information on author contributions (Additional file 1). Authors are usually asked to briefly describe which task was conducted by which co-author. Although this information is valuable, it does not provide an objective, straightforward and universal measure of author contribution. For example, ‘data collection’ for a review article may in some cases simply involve searching a database by filtering papers using specific key words, while it may be a very time-consuming task in field ecology, and a highly technical task in computational ecology. So ‘data collection’ can mean very different things depending on the field of study or the type of paper. In addition, individual tasks are often conducted by multiple authors but there is no way of knowing whether and to what extent one author has contributed more to them. Although some authorship contribution systems propose graded contributions for each task (e.g. lead, equal, supporting role in the CRedIT system [7], or the three-tier criterion proposed by the International Committee of Medical Journal Editor [8]), the lack of a quantitative value means these systems lack accuracy and produce qualitative data that is challenging to analyse or compare. The second common limitation is the complexity of the proposed metrics, which deters authors from providing data in the first place. A third major issue is the lack of fairness where often the lead or corresponding author can unilaterally decide on the order of the co-authors and the description of their contribution.

To address these shortcomings, we propose an easy to apply, universally comparable and fair tool to measure and report author contribution.

### A simple and accurate measure: percentage contributions

Percentages are straightforward and can be universally applied independent of research field, the number of co-authors or the nature of the paper (e.g. experimental, review, perspective, etc.). Because the authors of a paper are the best placed to make a judgement call about the value of each contribution, it is essential that percentage contributions are determined by authors rather than by a model based solely on the authors’ rank. Although disagreement may occur between co-authors, clarifying contribution among co-authors in the early stages of the research process is likely to ease potential tension [9] and in some cases prompt ‘real collaboration’. We propose that co-authors discuss and agree on their respective contributions prior to submitting their manuscript and these figures be provided by the corresponding author at the submission stage. By confirming their authorship, all co- authors confirm their agreement with their contributions and that of all other authors. This ensures that every published paper displays percentage contributions that have been discussed and agreed upon by every co-author.

A number of guidelines and best practices have been proposed for authors’ contributions (e.g. [10]). A possible starting point is to divide 100% by the number of authors and then estimate whether and to what extent each author provided more or less work than the others. The use of author-provided percentages has been proposed before to reflect the contribution of co-authors accurately (e.g. [2]) but with limited guidance about how to implement it. Verhagen et al. [11] developed the Quantitative Uniform Authorship Declaration (QUAD) approach, where each author is attributed percentage contributions in four categories: Conception and design, data collection, data analysis and conclusion and manuscript preparation. More recently, a very similar approach was proposed, based on scores rather than percentages with the more specific aim of deciding which contributor deserves authorship and which does not [12]. Clement [13] also suggests the use of four categories, albeit slightly different ones (ideas, work, writing and stewardship). However, an overly complicated metric may deter authors from applying it, and the criteria used in calculations must be consistent or have comparable importance across research fields, which may not be applicable to every type of article. In addition, many authors suggest that contributions should be restricted to an arbitrary threshold, for example 50% of the average contribution [13], 10% of the total work [11] or a threshold chosen by the authors [12]. Such limitation is likely to introduce major inconsistencies between papers, journals and fields of research, thereby preventing comparison. In addition, these thresholds limit the number of co-authors, which may affect interdisciplinary research and act as incentives to leave out minor contributors, potentially increasing ghost authorship (i.e. the omission of collaborators who did contribute to the work).

**Fig. 1:**
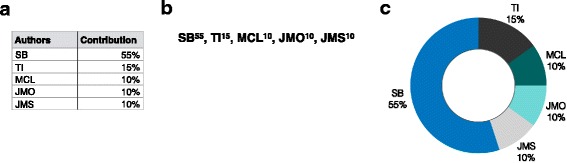
Examples of a table (**a**), text (**b**) or figure (**c**) that could be used to display author contribution percentages for a given paper. Data correspond to author contributions for the current paper

We propose that the contribution of each co-author be summarised in one number which must be more than 0% and less than 100% in multiple-authored papers. This provides a metric that is simpler for authors to determine and for the readers to grasp. In addition, this single metric imposes no upper limit on the number of authors. The percentage contribution should be displayed on the published paper either as raw numbers or as a figure (Fig. 1).

### A universally comparable metric: percentage-based author contribution index (ACI)

An outstanding limitation of percentage contributions is that they are difficult to compare across different papers because with more co-authors, it is mathematically difficult to obtain high percentages. As a consequence, author contributions cannot be directly compared between articles with unequal numbers of authors. To allow such comparisons, we propose a universal metric that takes into account the number of co-authors: the Author Contribution Index (ACI), calculated from the percentage contribution as per Eq. (1).

1 $$ \kern0.5em \mathrm{ACI}(i)= Ci\times \frac{n-1}{1- Ci} $$

where for author *i*,

*Ci* = contribution of author *i* in percentage (must be > 0 and < 1)

*n* = total number of authors including *i* (must be > 1).

ACI(*i*) reflects the contribution of author *i* as compared to the average contribution of all other authors. It is superior to one when the contribution of author *i* is larger than the average contribution of all other authors and inferior to one when the contribution of author *i* is less than the average contribution of all other authors. For example, on a paper written by three authors, where author *i* contributed 60% of the paper, ACI(*i*) = 3, meaning that author *i* contributed three times more than what the other authors contributed on average. Another useful metric is log_10_(ACI), which is positive when the author’s contribution is larger than the average contribution of all other authors and negative when the author’s contribution is less than the average. This metric is particularly useful to normalise data for further comparison and statistical analyses.

**Fig. 2:**
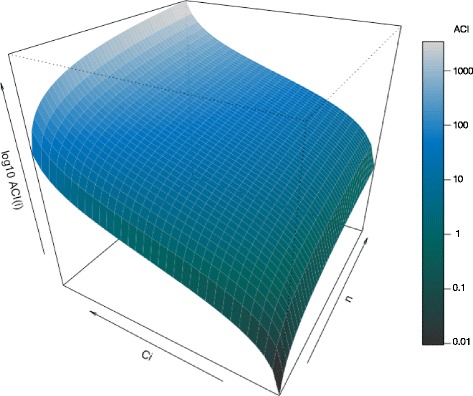
Universe of possible ACIs. X-axis: total number of authors including (*n* = 2 to *n* = 200); Y-axis: percentage contribution of author (=0.001 to =0.999); Z-axis: author contribution index for author (see Eq. (1)). Colours correspond to the value of ACI (see coloured scale on the right)

The graph in Fig. 2 displays the universe of all possible ACIs for papers written by up to 200 co-authors. The contribution profile of a particular author can be displayed in the universe of possible ACIs by adding dots, each representing one paper from the author being analysed. From these data, author profiles may appear according to a variety of criteria such as time, author’s seniority, area of research and type of institution where the author works, among others. Based on Eq. (1), it is also possible to calculate average ACI for an individual author or to plot the ACI frequency distribution of an individual author based on all or specific parts of his publications.

**Fig. 3:**
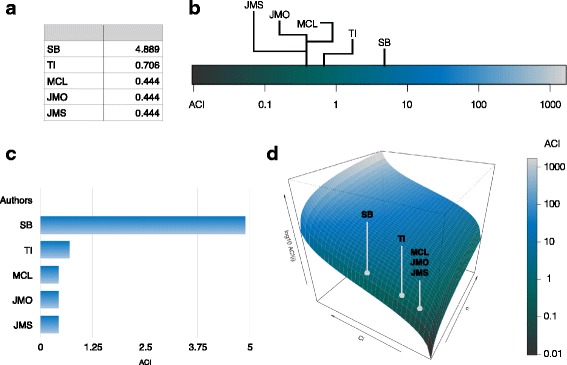
Examples of table (**a**) or figures (**b, c, d**) that could be used to display the author contribution index (ACI) for a given paper. Data corresponds to author contributions for the current paper

One notable feature of ACI is that it increases with the proportion of work produced but also with the number of ‘minor’ co-authors (Fig. 2). By giving more weight to main contributors of papers with many co-authors, ACI recognises the skills required and work involved in leading large collaborative projects. Figure 3 provides examples of how ACIs could be displayed in a paper.

### A fair tool: assisting job seekers, recruiters and performance-based evaluations

The scientific community seems to have reached the consensus that journal impact factors are not an accurate measure of the value of a particular article or the value of its author(s) [14]. One of the main reasons is that a very highly ranked journal may publish few articles that are heavily cited, but it may also publish a number of papers that have very little impact. In recent years, article-based impact has been preferred to journal impact factor. For example, the Hirsch index (h-index), which is based on the number of citations of one’s papers, is now widely used to gauge the output of a scientist. However, the h-index can also be manipulated [15] and it does not provide a measure of the amount of work produced by each co-author, which means guest or honorary authorship (i.e. inclusion of authors who did not contribute to the work) cannot be accounted for. This issue of guest authorship has been denounced widely in medical and clinical science [16], but other research fields are not immune to the problem (e.g. in ecology [17], environmental science [18], geography [19], geology [20], etc.). Since genuine authors suffer no cost when they add co-authors, papers tend to have more and more co-authors [21, 22]. With percentage contributions, the amount of work invested in a paper is a finite value (100%). Therefore, when more authors are added as a ‘gift’, they all need to be attributed a percentage of the work. In this zero-sum game, either it will be visible that guest authors have contributed an extremely small proportion of the work—and should receive very little recognition—or the genuine authors will have to give away large chunks of their well-deserved credit.

To ensure fair values are reported, we propose that co-authors discuss and agree on their respective contributions prior to submitting their manuscript and these figures be provided by the corresponding author at the submission stage. By confirming authorship, all co-authors confirm their agreement with their contributions and that of all other authors. This ensures that every published paper displays percentage contributions that have been discussed and agreed upon by every co-author.

The sentence-based descriptions of authors’ contribution that are used by some journals provide an indication of tasks performed by each author. Maintaining and generalising this practice as a detailed record of the role of each author is essential to increasing transparency. However, obtaining a clear idea of the amount of work a scientist is actually providing is difficult if one needs to read through all the authors’ contribution sections and weigh in the topic, the type of paper, the number of co-authors, etc. The proposed index has the potential to complement descriptive authorship information sections with a quantitative measure, which is much easier to analyse, summarise and compare across a large number of publications. For example, ACI can help in sifting through the numerous papers published by one scientist to select only those where this particular author has made a major contribution. This short list of papers could then be analysed in more details, for example using the written author contribution sections.

ACI can provide valuable information for performance-based evaluation processes and could be implemented in existing reporting systems. This includes internal evaluation for career advancement, as well as research productivity evaluation for funding purposes and national-scale ranking schemes (such as the Performance-based Research Fund (PBRF) system in New Zealand or the Research Excellence Framework (REF) in the UK). It is also in the advantage of the candidate to be able to demonstrate his/her actual contribution to a potential recruiter who may ask ‘What have you done on all these papers listed on your CV?’ One could answer such a question by analysing the distribution of a scientist’s ACI and its evolution through time or by calculating and comparing his/her average ACIs in experimental, review and perspective papers. ACI could also be used as an additional metric in network-based collaboration analyses (e.g. [23]) or to further inform composite citation indicators (e.g. [24]).

### A first look: testing ACI

#### Aims

Here, we provide an analysis of ACI calculated from work published in the past 3 years (January 2014–December 2016) based on contribution percentages provided by scientists who volunteered to respond to an online survey (Additional file 2). This survey aims at (1) demonstrating that authors can provide percentage contribution for their work and are willing to do so, (2) demonstrating that there is an interest from the scientific community to provide more transparency in author contribution, (3) exemplifying basic calculations that can be easily performed using ACI values and (4) demonstrating that ACI can be used to understand the scientific production of scientists in relation to their career advancement. Because the contribution percentages were provided after publication and without discussion among co-authors, these values may not be as accurate as if they had been agreed upon by all co-authors prior to publication [10]. Hence, the aim of this exercise was not to produce a highly accurate dataset, and therefore, the following analysis should be regarded as illustrative.

## Methods

Respondents were asked to provide their percentage contributions for all the articles they co-authored in the past 3 years. Additional information collected was country of origin, time since research active and job description (Additional file 2). The URL address and information about the online survey were distributed through electronic mailing lists, ecological society newsletters and social media. Responses were collected between September 4, 2016, and January 8, 2017. During this timeframe, 97 ecology scientists from 19 different countries completed the survey. Respondents were anonymous and no filtering of respondents was performed other than removing incomplete entries. Data with contradictory or obviously inaccurate information (for example multi-authored publications where the respondent claims 100% of the work) were removed. The final dataset comprised data for 836 publications from 97 ecology researchers. Author contribution indices (ACIs) of the respondents were calculated for each publication using Eq. (1). ACIs were log-transformed (log_10_(ACI)) to meet the assumptions of normality and all statistical analyses were conducted in *R* [25]. Respondents were asked to provide information about the number of years they have been research active. This was defined as the time from first year of PhD study or first published peer-reviewed paper, whichever came first. Linear regression and *F* statistics were used to analyse ACI in relation to the number of years as an active researcher.

Respondents were categorised in different job positions as follows: postgrad, a postgraduate student; postdoc, a postdoctoral fellow or other non-permanent staff; ECR, a tenure or permanent early-career researcher; PI, a mid-career principal investigator; prof, an associate professor of full professor; other. ACI was analysed in relation to job description using ANOVA with a priori contrasts between non-permanent supervised staff (postgraduate and postdoc) and permanent and independent researchers (ECR, PI and professors), as well as between all early career researchers (postgraduate, postdoc and ECR) and established researchers (mid-career PI and professors).

## Results and discussion

**Fig. 4:**
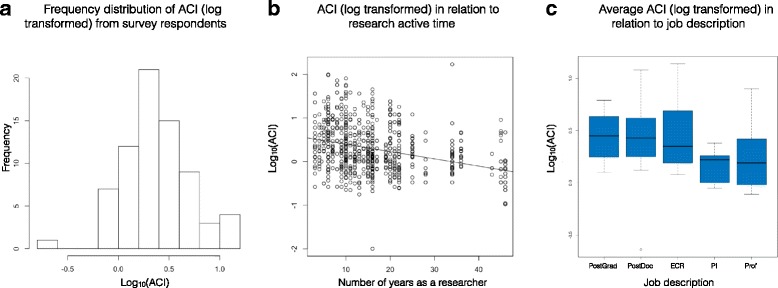
Descriptive statistics of author contribution index (ACI, see Eq. (1)) Data from an online anonymous survey completed by 97 authors between 2014 and 2016. **a** Frequency distribution of ACI (log transformed) from survey respondents. **b** ACI (log transformed) in relation to research active time. **c** Average ACI (log transformed) in relation to job description

ACI varied from 0.0101, which means the author claims to have produced 101 times less work than his/her co-authors have on average, to 168, which the author claims to have produced 168 times more work than his co-authors have on average (Fig. 4a). Most researchers produced papers with a range of ACI values. Individuals with a majority of high ACI are likely to be drivers of publications, while those with a majority of medium ACI can be regarded as highly collaborative and those with a majority of low ACI may be service providers. The latter may provide assistance with a limited but potentially essential aspect of the research such as sampling, statistical treatment of the data, supervision or mentoring.

In our dataset, ACI varies in relation to the number of years as a researcher (F_1,835_ = 87.57, *p* < 0.0001); however, the correlation remains weak (*r* = 0.306), due to wide variability in ACI (Fig. 4c). With regards to job descriptions, average ACI was higher for non-permanent supervised staff (postgraduate students and postdoctoral fellows) compared to permanent and independent researchers (early career researchers, mid-career principal investigators and professors) (ANOVA *t* = 2.327, *p* = 0.0229). A similar difference was observed when comparing all early career researchers (post-graduate students, postdoc and early career researchers) to established researchers (mid-career principal investigators and Professors) (ANOVA *t* = 2.546, *p* = 0.0132) (Fig. 4). As researchers become independent and establish their own research team, they probably start supervising their own students and postdocs and their ACI is likely to decrease accordingly.

It is possible to link ACI with article impact through the number of citations, altmetrics or any other article-based impact metrics, for example, by dividing ACI by the number of citations for a particular paper. By averaging ACIs, one could also summarise the output of a given scientist as a single number as suggested with other indices (e.g. [26]). However, we do not recommend such practice as it would largely mask the scientist’s output profile, thereby deceiving the purpose of ACI, and it would not be a meaningful way to compare scientist outputs as scientists with very different profiles may reach a very similar average ACI. In our dataset, there are numerous cases in which individuals at very different stages of their career reached a similar average ACI (Fig. 4, Additional file 3).

### Limitations and alternative applications

The proposed model requires authors to score precisely their contribution but does not provide a framework for making decisions around the value brought by each co-author. The focus of ACI is on the reporting and dissemination of a quantitative, accumulative and comparable measure of their contributions, after authors have agreed on their percentage contributions. The way these contributions are calculated and agreed upon is likely to vary greatly from one paper to another and does not equate to ‘time spent working on the paper’. For example, tasks requiring specific skills or expertise that are essential to the realisation of the work may rank highly, despite not being the most time-consuming. This apparent subjectivity should, however, be put in perspective with that of existing ranking systems. In any ranking systems, the reader can only place faith in the authors’ good judgement and ethical practices. Authors are the best placed to judge contribution to their own work and must be given the freedom, flexibility and adaptability to choose the percentage contributions that they believe are the most appropriate on a case by case basis.

It is important to note that the issues of arbitrary decisions and unethical behaviour, which may often arise from power dynamics between co-authors (e.g. between senior and junior scientists), will not be immediately resolved by using ACI. However, because percentage contributions have to be ratified by every co-author, groups are compelled to collegially discuss and score their contribution prior to submission, something that usually does not happen. It is expected that the mere fact of openly discussing contribution will reduce unilateral decisions and help to obtain thoughtful and more accurate contribution scores. With increased transparency, both among co-authors and between authors and readers, ethically questionable behaviours may not disappear, but they will become less common and easier to detect and report.

Another crucial limitation of ACI lies in the fact that a critical mass of data is required for the proposed metric to reach its true potential. Yet, its implementation by a significant number of journals or publishers will take a long time, if it is adopted at all. To circumvent this limitation and also generate ACI data from past literature and papers published in journals that will not implement the index, we propose that reference list repositories such as *ResearchGate* (researchgate.net) or *ORCID* (orcid.org) could provide an option for authors to record their percentage contributions to their publications. Because these values may not be vetted by all co-authors (as opposed to percentage contributions provided at the time of submission), several levels of verification should be displayed for each paper. Percentage contributions could be considered as (1) unverified if only one co-author provides them, (2) partially verified if at least a second co-author confirms the numbers and (3) fully verified if all co-authors of a paper confirm the numbers. In addition, we suggest authors to provide percentage contributions in their mansucripts, even if these are not specifically requested by the journal they publish in.

## Conclusion

There are many examples of author contribution indices that have been proposed but none has really been adopted by scientific journals. Many of the proposed solutions are either too complicated, not accurate enough or not comparable across articles, authors and disciplines. The author contribution index presented here addresses these three major issues and if adopted by scientific journals, it could significantly clarify the contribution of co-authors. This index is currently implemented in the recently launched journal *Rethinking Ecology* [3]. We hope that ACI will be adopted by many other journals to increase transparency in co-authored work and attribute accurate credit to authors. Although the current paper uses ecology as the focus, the proposed index is readily applicable to other scientific fields. Ambiguity around co-author contribution is a systemic issue in scientific publication, and accurate reporting of author contribution would benefit a very large variety of scientists in all fields.

The current paper focuses on the reporting of one percentage contribution and one ACI per paper and per author. Another possibility is to provide percentage contributions (and calculate ACI) for a variety of specific tasks. This would provide even more detailed information by specifying ‘who did how much of what’ on a given paper. However, task-specific ACI would only be useful if the same set of tasks is systematically reported, as proposed in the QUAD or the CReDIT approach, to allow comparisons between papers and meta-analyses.

The proposed ACI index has the potential to contribute to more transparency in the science literature, and if adopted by scientists, it could provide job seekers, recruiters and evaluating bodies with a tool to gather information that is essential to them and cannot be easily and accurately obtained otherwise.

## Additional files

Additional file 1: Journal’s policy on authors’ contribution section for the top 150 ecology journals (according to ISI Web of Knowledge). (DOCX 36 kb)

Additional file 2: Co-authorship survey used to collect data from ecology scientists. (DOCX 23 kb)

Additional file 3: Raw data obtained from the online co-authorship survey. (CSV 77 kb)
