# Supplementary material for: Percentage-based Author Contribution Index: a universal measure of author contribution to scientific articles
Source: Res Integr Peer Rev. 2017 Nov 3;2:18. doi: 10.1186/s41073-017-0042-y (PMC5803580; doi:10.1186/s41073-017-0042-y)
Supplement: Supplementary file 1 — Journal’s policy on authors’ contribution section for the top 150 ecology journals (according to ISI Web of Knowledge). (DOCX 36 kb) [file 41073_2017_42_MOESM1_ESM.docx]

**Supplementary Material 1. Journals policy on authors’ contribution section for the top 150 Ecology journals (according to ISI Web of Knowledge)**

| Abbreviated Journal Title | ISSN | Authors contribution section | |
| --- | --- | --- | --- |
|  |  |  |  |
|  |  |  |  |
|  |  | yes | no |
| [TRENDS ECOL EVOL](http://admin-apps.webofknowledge.com.ezproxy.lincoln.ac.nz/JCR/JCR?RQ=RECORD&rank=1&journal=TRENDS+ECOL+EVOL) | 0169-5347 |  | x |
| [ECOL LETT](http://admin-apps.webofknowledge.com.ezproxy.lincoln.ac.nz/JCR/JCR?RQ=RECORD&rank=2&journal=ECOL+LETT) | 1461-023X | x |  |
| [ANNU REV ECOL EVOL S](http://admin-apps.webofknowledge.com.ezproxy.lincoln.ac.nz/JCR/JCR?RQ=RECORD&rank=3&journal=ANNU+REV+ECOL+EVOL+S) | 1543-592X |  | x |
| [ISME J](http://admin-apps.webofknowledge.com.ezproxy.lincoln.ac.nz/JCR/JCR?RQ=RECORD&rank=4&journal=ISME+J) | 1751-7362 |  | x |
| [FRONT ECOL ENVIRON](http://admin-apps.webofknowledge.com.ezproxy.lincoln.ac.nz/JCR/JCR?RQ=RECORD&rank=5&journal=FRONT+ECOL+ENVIRON) | 1540-9295 |  | x |
| [GLOBAL CHANGE BIOL](http://admin-apps.webofknowledge.com.ezproxy.lincoln.ac.nz/JCR/JCR?RQ=RECORD&rank=6&journal=GLOBAL+CHANGE+BIOL) | 1354-1013 |  | x |
| [ECOL MONOGR](http://admin-apps.webofknowledge.com.ezproxy.lincoln.ac.nz/JCR/JCR?RQ=RECORD&rank=7&journal=ECOL+MONOGR) | 0012-9615 |  | x |
| [METHODS ECOL EVOL](http://admin-apps.webofknowledge.com.ezproxy.lincoln.ac.nz/JCR/JCR?RQ=RECORD&rank=8&journal=METHODS+ECOL+EVOL) | 2041-210X |  | x |
| [J ECOL](http://admin-apps.webofknowledge.com.ezproxy.lincoln.ac.nz/JCR/JCR?RQ=RECORD&rank=9&journal=J+ECOL) | 0022-0477 |  | x |
| [B AM MUS NAT HIST](http://admin-apps.webofknowledge.com.ezproxy.lincoln.ac.nz/JCR/JCR?RQ=RECORD&rank=10&journal=B+AM+MUS+NAT+HIST) | 0003-0090 |  | x |
| [MOL ECOL](http://admin-apps.webofknowledge.com.ezproxy.lincoln.ac.nz/JCR/JCR?RQ=RECORD&rank=11&journal=MOL+ECOL) | 0962-1083 | x |  |
| [GLOBAL ECOL BIOGEOGR](http://admin-apps.webofknowledge.com.ezproxy.lincoln.ac.nz/JCR/JCR?RQ=RECORD&rank=12&journal=GLOBAL+ECOL+BIOGEOGR) | 1466-822X |  | x |
| [ECOGRAPHY](http://admin-apps.webofknowledge.com.ezproxy.lincoln.ac.nz/JCR/JCR?RQ=RECORD&rank=13&journal=ECOGRAPHY) | 0906-7590 |  | x |
| [MOL ECOL RESOUR](http://admin-apps.webofknowledge.com.ezproxy.lincoln.ac.nz/JCR/JCR?RQ=RECORD&rank=14&journal=MOL+ECOL+RESOUR) | 1755-098X | x |  |
| [FUNCT ECOL](http://admin-apps.webofknowledge.com.ezproxy.lincoln.ac.nz/JCR/JCR?RQ=RECORD&rank=15&journal=FUNCT+ECOL) | 0269-8463 |  | x |
| [J APPL ECOL](http://admin-apps.webofknowledge.com.ezproxy.lincoln.ac.nz/JCR/JCR?RQ=RECORD&rank=16&journal=J+APPL+ECOL) | 0021-8901 |  | x |
| [WILDLIFE MONOGR](http://admin-apps.webofknowledge.com.ezproxy.lincoln.ac.nz/JCR/JCR?RQ=RECORD&rank=17&journal=WILDLIFE+MONOGR) | 0084-0173 |  | x |
| [J ANIM ECOL](http://admin-apps.webofknowledge.com.ezproxy.lincoln.ac.nz/JCR/JCR?RQ=RECORD&rank=18&journal=J+ANIM+ECOL) | 0021-8790 | x |  |
| [P ROY SOC B-BIOL SCI](http://admin-apps.webofknowledge.com.ezproxy.lincoln.ac.nz/JCR/JCR?RQ=RECORD&rank=19&journal=P+ROY+SOC+B-BIOL+SCI) | 0962-8452 | x |  |
| [ECOLOGY](http://admin-apps.webofknowledge.com.ezproxy.lincoln.ac.nz/JCR/JCR?RQ=RECORD&rank=20&journal=ECOLOGY) | 0012-9658 |  | x |
| [DIVERS DISTRIB](http://admin-apps.webofknowledge.com.ezproxy.lincoln.ac.nz/JCR/JCR?RQ=RECORD&rank=21&journal=DIVERS+DISTRIB) | 1366-9516 | x |  |
| [ECOSYST SERV](http://admin-apps.webofknowledge.com.ezproxy.lincoln.ac.nz/JCR/JCR?RQ=RECORD&rank=22&journal=ECOSYST+SERV) | 2212-0416 |  | x |
| [CONSERV BIOL](http://admin-apps.webofknowledge.com.ezproxy.lincoln.ac.nz/JCR/JCR?RQ=RECORD&rank=23&journal=CONSERV+BIOL) | 0888-8892 |  | x |
| [ECOL APPL](http://admin-apps.webofknowledge.com.ezproxy.lincoln.ac.nz/JCR/JCR?RQ=RECORD&rank=24&journal=ECOL+APPL) | 1051-0761 |  | x |
| [EVOLUTION](http://admin-apps.webofknowledge.com.ezproxy.lincoln.ac.nz/JCR/JCR?RQ=RECORD&rank=25&journal=EVOLUTION) | 0014-3820 |  | x |
| [J BIOGEOGR](http://admin-apps.webofknowledge.com.ezproxy.lincoln.ac.nz/JCR/JCR?RQ=RECORD&rank=26&journal=J+BIOGEOGR) | 0305-0270 | x |  |
| [BIOL CONSERV](http://admin-apps.webofknowledge.com.ezproxy.lincoln.ac.nz/JCR/JCR?RQ=RECORD&rank=27&journal=BIOL+CONSERV) | 0006-3207 |  | x |
| [ADV ECOL RES](http://admin-apps.webofknowledge.com.ezproxy.lincoln.ac.nz/JCR/JCR?RQ=RECORD&rank=28&journal=ADV+ECOL+RES) | 0065-2504 |  | x |
| [HEREDITY](http://admin-apps.webofknowledge.com.ezproxy.lincoln.ac.nz/JCR/JCR?RQ=RECORD&rank=29&journal=HEREDITY) | 0018-067X |  | x |
| [ECOSYSTEMS](http://admin-apps.webofknowledge.com.ezproxy.lincoln.ac.nz/JCR/JCR?RQ=RECORD&rank=30&journal=ECOSYSTEMS) | 1432-9840 | x |  |
| [BIOGEOSCIENCES](http://admin-apps.webofknowledge.com.ezproxy.lincoln.ac.nz/JCR/JCR?RQ=RECORD&rank=31&journal=BIOGEOSCIENCES) | 1726-4170 | x |  |
| [LANDSCAPE ECOL](http://admin-apps.webofknowledge.com.ezproxy.lincoln.ac.nz/JCR/JCR?RQ=RECORD&rank=32&journal=LANDSCAPE+ECOL) | 0921-2973 |  | x |
| [LANDSCAPE URBAN PLAN](http://admin-apps.webofknowledge.com.ezproxy.lincoln.ac.nz/JCR/JCR?RQ=RECORD&rank=33&journal=LANDSCAPE+URBAN+PLAN) | 0169-2046 |  | x |
| [OIKOS](http://admin-apps.webofknowledge.com.ezproxy.lincoln.ac.nz/JCR/JCR?RQ=RECORD&rank=34&journal=OIKOS) | 0030-1299 |  | x |
| [PERSPECT PLANT ECOL](http://admin-apps.webofknowledge.com.ezproxy.lincoln.ac.nz/JCR/JCR?RQ=RECORD&rank=35&journal=PERSPECT+PLANT+ECOL) | 1433-8319 |  | x |
| [AGR ECOSYST ENVIRON](http://admin-apps.webofknowledge.com.ezproxy.lincoln.ac.nz/JCR/JCR?RQ=RECORD&rank=36&journal=AGR+ECOSYST+ENVIRON) | 0167-8809 |  | x |
| [MICROB ECOL](http://admin-apps.webofknowledge.com.ezproxy.lincoln.ac.nz/JCR/JCR?RQ=RECORD&rank=37&journal=MICROB+ECOL) | 0095-3628 |  | x |
| [ECOL ECON](http://admin-apps.webofknowledge.com.ezproxy.lincoln.ac.nz/JCR/JCR?RQ=RECORD&rank=38&journal=ECOL+ECON) | 0921-8009 |  | x |
| [J CHEM ECOL](http://admin-apps.webofknowledge.com.ezproxy.lincoln.ac.nz/JCR/JCR?RQ=RECORD&rank=39&journal=J+CHEM+ECOL) | 0098-0331 |  | x |
| [J VEG SCI](http://admin-apps.webofknowledge.com.ezproxy.lincoln.ac.nz/JCR/JCR?RQ=RECORD&rank=40&journal=J+VEG+SCI) | 1100-9233 |  | x |
| [AM NAT](http://admin-apps.webofknowledge.com.ezproxy.lincoln.ac.nz/JCR/JCR?RQ=RECORD&rank=41&journal=AM+NAT) | 0003-0147 |  | x |
| [BEHAV ECOL](http://admin-apps.webofknowledge.com.ezproxy.lincoln.ac.nz/JCR/JCR?RQ=RECORD&rank=42&journal=BEHAV+ECOL) | 1045-2249 |  | x |
| [PALEOBIOLOGY](http://admin-apps.webofknowledge.com.ezproxy.lincoln.ac.nz/JCR/JCR?RQ=RECORD&rank=43&journal=PALEOBIOLOGY) | 0094-8373 |  | x |
| [OECOLOGIA](http://admin-apps.webofknowledge.com.ezproxy.lincoln.ac.nz/JCR/JCR?RQ=RECORD&rank=44&journal=OECOLOGIA) | 0029-8549 | x |  |
| [ECOL SOC](http://admin-apps.webofknowledge.com.ezproxy.lincoln.ac.nz/JCR/JCR?RQ=RECORD&rank=45&journal=ECOL+SOC) | 1708-3087 |  | x |
| [BIOL INVASIONS](http://admin-apps.webofknowledge.com.ezproxy.lincoln.ac.nz/JCR/JCR?RQ=RECORD&rank=46&journal=BIOL+INVASIONS) | 1387-3547 |  | x |
| [BIOL LETTERS](http://admin-apps.webofknowledge.com.ezproxy.lincoln.ac.nz/JCR/JCR?RQ=RECORD&rank=47&journal=BIOL+LETTERS) | 1744-9561 | x |  |
| [ANIM CONSERV](http://admin-apps.webofknowledge.com.ezproxy.lincoln.ac.nz/JCR/JCR?RQ=RECORD&rank=48&journal=ANIM+CONSERV) | 1367-9430 |  | x |
| [J EVOLUTION BIOL](http://admin-apps.webofknowledge.com.ezproxy.lincoln.ac.nz/JCR/JCR?RQ=RECORD&rank=49&journal=J+EVOLUTION+BIOL) | 1010-061X |  | x |
| [ECOL ENG](http://admin-apps.webofknowledge.com.ezproxy.lincoln.ac.nz/JCR/JCR?RQ=RECORD&rank=50&journal=ECOL+ENG) | 0925-8574 |  | x |
| [BMC ECOL](http://admin-apps.webofknowledge.com.ezproxy.lincoln.ac.nz/JCR/JCR?RQ=RECORD&rank=51&journal=BMC+ECOL) | 1472-6785 | x |  |
| [CURR OPIN INSECT SCI](http://admin-apps.webofknowledge.com.ezproxy.lincoln.ac.nz/JCR/JCR?RQ=RECORD&rank=52&journal=CURR+OPIN+INSECT+SCI) | 2214-5745 |  | x |
| [FUNGAL ECOL](http://admin-apps.webofknowledge.com.ezproxy.lincoln.ac.nz/JCR/JCR?RQ=RECORD&rank=53&journal=FUNGAL+ECOL) | 1754-5048 |  | x |
| [ECOL EVOL](http://admin-apps.webofknowledge.com.ezproxy.lincoln.ac.nz/JCR/JCR?RQ=RECORD&rank=54&journal=ECOL+EVOL) | 2045-7758 |  | x |
| [FRESHW SCI](http://admin-apps.webofknowledge.com.ezproxy.lincoln.ac.nz/JCR/JCR?RQ=RECORD&rank=55&journal=FRESHW+SCI) | 2161-9549 |  | x |
| [BEHAV ECOL SOCIOBIOL](http://admin-apps.webofknowledge.com.ezproxy.lincoln.ac.nz/JCR/JCR?RQ=RECORD&rank=56&journal=BEHAV+ECOL+SOCIOBIOL) | 0340-5443 |  | x |
| [MAR ECOL PROG SER](http://admin-apps.webofknowledge.com.ezproxy.lincoln.ac.nz/JCR/JCR?RQ=RECORD&rank=57&journal=MAR+ECOL+PROG+SER) | 0171-8630 |  | x |
| [ECOTOXICOLOGY](http://admin-apps.webofknowledge.com.ezproxy.lincoln.ac.nz/JCR/JCR?RQ=RECORD&rank=58&journal=ECOTOXICOLOGY) | 0963-9292 |  | x |
| [APPL VEG SCI](http://admin-apps.webofknowledge.com.ezproxy.lincoln.ac.nz/JCR/JCR?RQ=RECORD&rank=59&journal=APPL+VEG+SCI) | 1402-2001 |  | x |
| [ECOSPHERE](http://admin-apps.webofknowledge.com.ezproxy.lincoln.ac.nz/JCR/JCR?RQ=RECORD&rank=60&journal=ECOSPHERE) | 2150-8925 |  | x |
| [ECOL MODEL](http://admin-apps.webofknowledge.com.ezproxy.lincoln.ac.nz/JCR/JCR?RQ=RECORD&rank=61&journal=ECOL+MODEL) | 0304-3800 |  | x |
| [BIODIVERS CONSERV](http://admin-apps.webofknowledge.com.ezproxy.lincoln.ac.nz/JCR/JCR?RQ=RECORD&rank=62&journal=BIODIVERS+CONSERV) | 0960-3115 |  | x |
| [J NAT CONSERV](http://admin-apps.webofknowledge.com.ezproxy.lincoln.ac.nz/JCR/JCR?RQ=RECORD&rank=63&journal=J+NAT+CONSERV) | 1617-1381 |  | x |
| [ECOHYDROLOGY](http://admin-apps.webofknowledge.com.ezproxy.lincoln.ac.nz/JCR/JCR?RQ=RECORD&rank=64&journal=ECOHYDROLOGY) | 1936-0584 |  | x |
| [AQUAT MICROB ECOL](http://admin-apps.webofknowledge.com.ezproxy.lincoln.ac.nz/JCR/JCR?RQ=RECORD&rank=65&journal=AQUAT+MICROB+ECOL) | 0948-3055 | x |  |
| [THEOR ECOL-NETH](http://admin-apps.webofknowledge.com.ezproxy.lincoln.ac.nz/JCR/JCR?RQ=RECORD&rank=66&journal=THEOR+ECOL-NETH) | 1874-1738 |  | x |
| [ORYX](http://admin-apps.webofknowledge.com.ezproxy.lincoln.ac.nz/JCR/JCR?RQ=RECORD&rank=67&journal=ORYX) | 0030-6053 |  | x |
| [URBAN ECOSYST](http://admin-apps.webofknowledge.com.ezproxy.lincoln.ac.nz/JCR/JCR?RQ=RECORD&rank=68&journal=URBAN+ECOSYST) | 1083-8155 |  | x |
| [AQUAT INVASIONS](http://admin-apps.webofknowledge.com.ezproxy.lincoln.ac.nz/JCR/JCR?RQ=RECORD&rank=69&journal=AQUAT+INVASIONS) | 1798-6540 |  | x |
| [EUR J SOIL BIOL](http://admin-apps.webofknowledge.com.ezproxy.lincoln.ac.nz/JCR/JCR?RQ=RECORD&rank=70&journal=EUR+J+SOIL+BIOL) | 1164-5563 |  | x |
| [BIOTROPICA](http://admin-apps.webofknowledge.com.ezproxy.lincoln.ac.nz/JCR/JCR?RQ=RECORD&rank=71&journal=BIOTROPICA) | 0006-3606 |  | x |
| [RESTOR ECOL](http://admin-apps.webofknowledge.com.ezproxy.lincoln.ac.nz/JCR/JCR?RQ=RECORD&rank=72&journal=RESTOR+ECOL) | 1061-2971 | x |  |
| [EVOL ECOL](http://admin-apps.webofknowledge.com.ezproxy.lincoln.ac.nz/JCR/JCR?RQ=RECORD&rank=73&journal=EVOL+ECOL) | 0269-7653 |  | x |
| [CHEMOECOLOGY](http://admin-apps.webofknowledge.com.ezproxy.lincoln.ac.nz/JCR/JCR?RQ=RECORD&rank=74&journal=CHEMOECOLOGY) | 0937-7409 |  | x |
| [BASIC APPL ECOL](http://admin-apps.webofknowledge.com.ezproxy.lincoln.ac.nz/JCR/JCR?RQ=RECORD&rank=75&journal=BASIC+APPL+ECOL) | 1439-1791 |  | x |
| [PHYTOCOENOLOGIA](http://admin-apps.webofknowledge.com.ezproxy.lincoln.ac.nz/JCR/JCR?RQ=RECORD&rank=76&journal=PHYTOCOENOLOGIA) | 0340-269X | x |  |
| [AQUAT ECOL](http://admin-apps.webofknowledge.com.ezproxy.lincoln.ac.nz/JCR/JCR?RQ=RECORD&rank=77&journal=AQUAT+ECOL) | 1386-2588 |  | x |
| [ECOL COMPLEX](http://admin-apps.webofknowledge.com.ezproxy.lincoln.ac.nz/JCR/JCR?RQ=RECORD&rank=78&journal=ECOL+COMPLEX) | 1476-945X |  | x |
| [J EXP MAR BIOL ECOL](http://admin-apps.webofknowledge.com.ezproxy.lincoln.ac.nz/JCR/JCR?RQ=RECORD&rank=79&journal=J+EXP+MAR+BIOL+ECOL) | 0022-0981 |  | x |
| [J PLANT ECOL](http://admin-apps.webofknowledge.com.ezproxy.lincoln.ac.nz/JCR/JCR?RQ=RECORD&rank=80&journal=J+PLANT+ECOL) | 1752-9921 |  | x |
| [J SOIL WATER CONSERV](http://admin-apps.webofknowledge.com.ezproxy.lincoln.ac.nz/JCR/JCR?RQ=RECORD&rank=81&journal=J+SOIL+WATER+CONSERV) | 0022-4561 |  | x |
| [POLAR RES](http://admin-apps.webofknowledge.com.ezproxy.lincoln.ac.nz/JCR/JCR?RQ=RECORD&rank=82&journal=POLAR+RES) | 0800-0395 |  | x |
| [J WILDLIFE MANAGE](http://admin-apps.webofknowledge.com.ezproxy.lincoln.ac.nz/JCR/JCR?RQ=RECORD&rank=83&journal=J+WILDLIFE+MANAGE) | 0022-541X |  | x |
| [POLAR BIOL](http://admin-apps.webofknowledge.com.ezproxy.lincoln.ac.nz/JCR/JCR?RQ=RECORD&rank=84&journal=POLAR+BIOL) | 0722-4060 |  | x |
| [POPUL ECOL](http://admin-apps.webofknowledge.com.ezproxy.lincoln.ac.nz/JCR/JCR?RQ=RECORD&rank=85&journal=POPUL+ECOL) | 1438-3896 |  | x |
| [ECOL INFORM](http://admin-apps.webofknowledge.com.ezproxy.lincoln.ac.nz/JCR/JCR?RQ=RECORD&rank=86&journal=ECOL+INFORM) | 1574-9541 |  | x |
| [MAR BIOL RES](http://admin-apps.webofknowledge.com.ezproxy.lincoln.ac.nz/JCR/JCR?RQ=RECORD&rank=87&journal=MAR+BIOL+RES) | 1745-1000 |  | x |
| [S AFR J WILDL RES](http://admin-apps.webofknowledge.com.ezproxy.lincoln.ac.nz/JCR/JCR?RQ=RECORD&rank=88&journal=S+AFR+J+WILDL+RES) | 0379-4369 |  | x |
| [J ARID ENVIRON](http://admin-apps.webofknowledge.com.ezproxy.lincoln.ac.nz/JCR/JCR?RQ=RECORD&rank=89&journal=J+ARID+ENVIRON) | 0140-1963 |  | x |
| [INT J SUST DEV WORLD](http://admin-apps.webofknowledge.com.ezproxy.lincoln.ac.nz/JCR/JCR?RQ=RECORD&rank=90&journal=INT+J+SUST+DEV+WORLD) | 1350-4509 |  | x |
| [AUSTRAL ECOL](http://admin-apps.webofknowledge.com.ezproxy.lincoln.ac.nz/JCR/JCR?RQ=RECORD&rank=91&journal=AUSTRAL+ECOL) | 1442-9985 |  | x |
| [FLORA](http://admin-apps.webofknowledge.com.ezproxy.lincoln.ac.nz/JCR/JCR?RQ=RECORD&rank=92&journal=FLORA) | 0367-2530 |  | x |
| [PEDOBIOLOGIA](http://admin-apps.webofknowledge.com.ezproxy.lincoln.ac.nz/JCR/JCR?RQ=RECORD&rank=93&journal=PEDOBIOLOGIA) | 0031-4056 |  | x |
| [WETLANDS](http://admin-apps.webofknowledge.com.ezproxy.lincoln.ac.nz/JCR/JCR?RQ=RECORD&rank=94&journal=WETLANDS) | 0277-5212 |  | x |
| [PLANT ECOL](http://admin-apps.webofknowledge.com.ezproxy.lincoln.ac.nz/JCR/JCR?RQ=RECORD&rank=95&journal=PLANT+ECOL) | 1385-0237 |  | x |
| [THEOR POPUL BIOL](http://admin-apps.webofknowledge.com.ezproxy.lincoln.ac.nz/JCR/JCR?RQ=RECORD&rank=96&journal=THEOR+POPUL+BIOL) | 0040-5809 |  | x |
| [ACTA OECOL](http://admin-apps.webofknowledge.com.ezproxy.lincoln.ac.nz/JCR/JCR?RQ=RECORD&rank=97&journal=ACTA+OECOL) | 1146-609X |  | x |
| [ENVIRON BIOL FISH](http://admin-apps.webofknowledge.com.ezproxy.lincoln.ac.nz/JCR/JCR?RQ=RECORD&rank=98&journal=ENVIRON+BIOL+FISH) | 0378-1909 |  | x |
| [EUR J WILDLIFE RES](http://admin-apps.webofknowledge.com.ezproxy.lincoln.ac.nz/JCR/JCR?RQ=RECORD&rank=99&journal=EUR+J+WILDLIFE+RES) | 1612-4642 |  | x |
| [RANGELAND ECOL MANAG](http://admin-apps.webofknowledge.com.ezproxy.lincoln.ac.nz/JCR/JCR?RQ=RECORD&rank=100&journal=RANGELAND+ECOL+MANAG) | 1550-7424 |  | x |
| [ECOL RES](http://admin-apps.webofknowledge.com.ezproxy.lincoln.ac.nz/JCR/JCR?RQ=RECORD&rank=101&journal=ECOL+RES) | 0912-3814 |  | x |
| [PLANT SPEC BIOL](http://admin-apps.webofknowledge.com.ezproxy.lincoln.ac.nz/JCR/JCR?RQ=RECORD&rank=102&journal=PLANT+SPEC+BIOL) | 0913-557X |  | x |
| [CHEM ECOL](http://admin-apps.webofknowledge.com.ezproxy.lincoln.ac.nz/JCR/JCR?RQ=RECORD&rank=103&journal=CHEM+ECOL) | 0275-7540 |  | x |
| [AFR J RANGE FOR SCI](http://admin-apps.webofknowledge.com.ezproxy.lincoln.ac.nz/JCR/JCR?RQ=RECORD&rank=104&journal=AFR+J+RANGE+FOR+SCI) | 1022-0119 |  | x |
| [NEW ZEAL J ECOL](http://admin-apps.webofknowledge.com.ezproxy.lincoln.ac.nz/JCR/JCR?RQ=RECORD&rank=105&journal=NEW+ZEAL+J+ECOL) | 0110-6465 |  | x |
| [B PEABODY MUS NAT HI](http://admin-apps.webofknowledge.com.ezproxy.lincoln.ac.nz/JCR/JCR?RQ=RECORD&rank=106&journal=B+PEABODY+MUS+NAT+HI) | 0079-032X |  | x |
| [RANGELAND J](http://admin-apps.webofknowledge.com.ezproxy.lincoln.ac.nz/JCR/JCR?RQ=RECORD&rank=107&journal=RANGELAND+J) | 1036-9872 |  | x |
| [POL POLAR RES](http://admin-apps.webofknowledge.com.ezproxy.lincoln.ac.nz/JCR/JCR?RQ=RECORD&rank=108&journal=POL+POLAR+RES) | 0138-0338 |  | x |
| [TROP ECOL](http://admin-apps.webofknowledge.com.ezproxy.lincoln.ac.nz/JCR/JCR?RQ=RECORD&rank=109&journal=TROP+ECOL) | 0564-3295 |  | x |
| [POLAR SCI](http://admin-apps.webofknowledge.com.ezproxy.lincoln.ac.nz/JCR/JCR?RQ=RECORD&rank=110&journal=POLAR+SCI) | 1873-9652 |  | x |
| [J BIOL DYNAM](http://admin-apps.webofknowledge.com.ezproxy.lincoln.ac.nz/JCR/JCR?RQ=RECORD&rank=111&journal=J+BIOL+DYNAM) | 1751-3758 |  | x |
| [FIRE ECOL](http://admin-apps.webofknowledge.com.ezproxy.lincoln.ac.nz/JCR/JCR?RQ=RECORD&rank=112&journal=FIRE+ECOL) | 1933-9747 |  | x |
| [COMMUNITY ECOL](http://admin-apps.webofknowledge.com.ezproxy.lincoln.ac.nz/JCR/JCR?RQ=RECORD&rank=113&journal=COMMUNITY+ECOL) | 1585-8553 |  | x |
| [J NAT HIST](http://admin-apps.webofknowledge.com.ezproxy.lincoln.ac.nz/JCR/JCR?RQ=RECORD&rank=114&journal=J+NAT+HIST) | 0022-2933 |  | x |
| [WILDLIFE RES](http://admin-apps.webofknowledge.com.ezproxy.lincoln.ac.nz/JCR/JCR?RQ=RECORD&rank=115&journal=WILDLIFE+RES) | 1035-3712 |  | x |
| [BIOCHEM SYST ECOL](http://admin-apps.webofknowledge.com.ezproxy.lincoln.ac.nz/JCR/JCR?RQ=RECORD&rank=116&journal=BIOCHEM+SYST+ECOL) | 0305-1978 |  | x |
| [J TROP ECOL](http://admin-apps.webofknowledge.com.ezproxy.lincoln.ac.nz/JCR/JCR?RQ=RECORD&rank=117&journal=J+TROP+ECOL) | 0266-4674 |  | x |
| [WILDLIFE BIOL](http://admin-apps.webofknowledge.com.ezproxy.lincoln.ac.nz/JCR/JCR?RQ=RECORD&rank=118&journal=WILDLIFE+BIOL) | 0909-6396 |  | x |
| [P ACAD NAT SCI PHILA](http://admin-apps.webofknowledge.com.ezproxy.lincoln.ac.nz/JCR/JCR?RQ=RECORD&rank=119&journal=P+ACAD+NAT+SCI+PHILA) | 0097-3157 |  | x |
| [POLAR REC](http://admin-apps.webofknowledge.com.ezproxy.lincoln.ac.nz/JCR/JCR?RQ=RECORD&rank=120&journal=POLAR+REC) | 0032-2474 |  | x |
| [AFR J ECOL](http://admin-apps.webofknowledge.com.ezproxy.lincoln.ac.nz/JCR/JCR?RQ=RECORD&rank=121&journal=AFR+J+ECOL) | 0141-6707 |  | x |
| [J FISH WILDL MANAG](http://admin-apps.webofknowledge.com.ezproxy.lincoln.ac.nz/JCR/JCR?RQ=RECORD&rank=122&journal=J+FISH+WILDL+MANAG) | 1944-687X |  | x |
| [ANN ZOOL FENN](http://admin-apps.webofknowledge.com.ezproxy.lincoln.ac.nz/JCR/JCR?RQ=RECORD&rank=123&journal=ANN+ZOOL+FENN) | 0003-455X |  | x |
| [REV CHIL HIST NAT](http://admin-apps.webofknowledge.com.ezproxy.lincoln.ac.nz/JCR/JCR?RQ=RECORD&rank=124&journal=REV+CHIL+HIST+NAT) | 0716-078X | x |  |
| [ISR J ECOL EVOL](http://admin-apps.webofknowledge.com.ezproxy.lincoln.ac.nz/JCR/JCR?RQ=RECORD&rank=125&journal=ISR+J+ECOL+EVOL) | 1565-9801 |  | x |
| [J FRESHWATER ECOL](http://admin-apps.webofknowledge.com.ezproxy.lincoln.ac.nz/JCR/JCR?RQ=RECORD&rank=126&journal=J+FRESHWATER+ECOL) | 0270-5060 |  | x |
| [NAT AREA J](http://admin-apps.webofknowledge.com.ezproxy.lincoln.ac.nz/JCR/JCR?RQ=RECORD&rank=127&journal=NAT+AREA+J) | 0885-8608 |  | x |
| [LANDSC ECOL ENG](http://admin-apps.webofknowledge.com.ezproxy.lincoln.ac.nz/JCR/JCR?RQ=RECORD&rank=128&journal=LANDSC+ECOL+ENG) | 1860-1871 |  | x |
| [ECOSCIENCE](http://admin-apps.webofknowledge.com.ezproxy.lincoln.ac.nz/JCR/JCR?RQ=RECORD&rank=129&journal=ECOSCIENCE) | 1195-6860 |  | x |
| [AM MIDL NAT](http://admin-apps.webofknowledge.com.ezproxy.lincoln.ac.nz/JCR/JCR?RQ=RECORD&rank=130&journal=AM+MIDL+NAT) | 0003-0031 |  | x |
| [EKOLOJI](http://admin-apps.webofknowledge.com.ezproxy.lincoln.ac.nz/JCR/JCR?RQ=RECORD&rank=131&journal=EKOLOJI) | 1300-1361 |  | x |
| [EVOL ECOL RES](http://admin-apps.webofknowledge.com.ezproxy.lincoln.ac.nz/JCR/JCR?RQ=RECORD&rank=132&journal=EVOL+ECOL+RES) | 1522-0613 |  | x |
| [NORTHEAST NAT](http://admin-apps.webofknowledge.com.ezproxy.lincoln.ac.nz/JCR/JCR?RQ=RECORD&rank=133&journal=NORTHEAST+NAT) | 1092-6194 |  | x |
| [COMPOST SCI UTIL](http://admin-apps.webofknowledge.com.ezproxy.lincoln.ac.nz/JCR/JCR?RQ=RECORD&rank=134&journal=COMPOST+SCI+UTIL) | 1065-657X |  | x |
| [SOUTHEAST NAT](http://admin-apps.webofknowledge.com.ezproxy.lincoln.ac.nz/JCR/JCR?RQ=RECORD&rank=135&journal=SOUTHEAST+NAT) | 1528-7092 |  | x |
| [APPL ECOL ENV RES](http://admin-apps.webofknowledge.com.ezproxy.lincoln.ac.nz/JCR/JCR?RQ=RECORD&rank=136&journal=APPL+ECOL+ENV+RES) | 1589-1623 |  | x |
| [POL J ECOL](http://admin-apps.webofknowledge.com.ezproxy.lincoln.ac.nz/JCR/JCR?RQ=RECORD&rank=137&journal=POL+J+ECOL) | 1505-2249 |  | x |
| [RUSS J ECOL+](http://admin-apps.webofknowledge.com.ezproxy.lincoln.ac.nz/JCR/JCR?RQ=RECORD&rank=138&journal=RUSS+J+ECOL%2B) | 1067-4136 |  | x |
| [NORTHWEST SCI](http://admin-apps.webofknowledge.com.ezproxy.lincoln.ac.nz/JCR/JCR?RQ=RECORD&rank=139&journal=NORTHWEST+SCI) | 0029-344X |  | x |
| [ACTA AMAZON](http://admin-apps.webofknowledge.com.ezproxy.lincoln.ac.nz/JCR/JCR?RQ=RECORD&rank=140&journal=ACTA+AMAZON) | 0044-5967 |  | x |
| [ECOTROPICA](http://admin-apps.webofknowledge.com.ezproxy.lincoln.ac.nz/JCR/JCR?RQ=RECORD&rank=141&journal=ECOTROPICA) | 0949-3026 |  | x |
| [WEST N AM NATURALIST](http://admin-apps.webofknowledge.com.ezproxy.lincoln.ac.nz/JCR/JCR?RQ=RECORD&rank=142&journal=WEST+N+AM+NATURALIST) | 1527-0904 |  | x |
| [ECO MONT](http://admin-apps.webofknowledge.com.ezproxy.lincoln.ac.nz/JCR/JCR?RQ=RECORD&rank=143&journal=ECO+MONT) | 2073-106X |  | x |
| [CONTEMP PROBL ECOL+](http://admin-apps.webofknowledge.com.ezproxy.lincoln.ac.nz/JCR/JCR?RQ=RECORD&rank=144&journal=CONTEMP+PROBL+ECOL%2B) | 1995-4255 |  | x |
| [SOUTHWEST NAT](http://admin-apps.webofknowledge.com.ezproxy.lincoln.ac.nz/JCR/JCR?RQ=RECORD&rank=145&journal=SOUTHWEST+NAT) | 0038-4909 |  | x |
| [VIE MILIEU](http://admin-apps.webofknowledge.com.ezproxy.lincoln.ac.nz/JCR/JCR?RQ=RECORD&rank=146&journal=VIE+MILIEU) | 0240-8759 |  | x |
| [INTERCIENCIA](http://admin-apps.webofknowledge.com.ezproxy.lincoln.ac.nz/JCR/JCR?RQ=RECORD&rank=147&journal=INTERCIENCIA) | 0378-1844 |  | x |
| [P LINN SOC N S W](http://admin-apps.webofknowledge.com.ezproxy.lincoln.ac.nz/JCR/JCR?RQ=RECORD&rank=148&journal=P+LINN+SOC+N+S+W) | 0370-047X |  | x |
| [REV ECOL-TERRE VIE](http://admin-apps.webofknowledge.com.ezproxy.lincoln.ac.nz/JCR/JCR?RQ=RECORD&rank=149&journal=REV+ECOL-TERRE+VIE) | 0249-7395 |  | x |
| [NAT HIST](http://admin-apps.webofknowledge.com.ezproxy.lincoln.ac.nz/JCR/JCR?RQ=RECORD&rank=150&journal=NAT+HIST) | 0028-0712 |  | x |
