# Supplementary material for: Percentage-based Author Contribution Index: a universal measure of author contribution to scientific articles
Source: Res Integr Peer Rev. 2017 Nov 3;2:18. doi: 10.1186/s41073-017-0042-y (PMC5803580; doi:10.1186/s41073-017-0042-y)
Supplement: Supplementary file 2 — Co-authorship survey used to collect data from ecology scientists. (DOCX 23 kb) [file 41073_2017_42_MOESM2_ESM.docx]

**Supplementary Material 2: Co-authorship survey used to collect data from ecology scientists**

The description below is a copy of the survey. The actual survey was only available online at the following address: <http://www.surveygizmo.com/s3/3024711/New-Survey>.

We are conducting a study on co-authorship among ecologists. In this questionnaire, you will be asked to provide information about your recently published papers (2014-2016). It may help to have your CV or list of publications at hand so you don't forget any papers. This questionnaire is anonymous. By answering you agree that the data you provide is used for the above-mentioned analysis and subsequent publication.

Which country are you based in?

How many years have you been research active (from first year of PhD study or first published peer-reviewed paper, whichever came first)?

Indicate whether you are:1) a postgraduate student, 2) a postdoctoral fellow or other non-permanent staff early career researcher (ECR), 3) a tenure or permanent ECR, 4) a mid-career principal investigator, or 5) an established Associate Professor of Full Professor.

How many peer-review articles have you published in the past 3 years, Including papers that are currently ‘In Press’?

For each paper you published in 2014-2016 (including papers currently 'In Press' ), provide the number of co-authors and the percentage of the work you have contributed. Please try to provide a fair and accurate estimation of your contribution. A good starting point is to divide 100% by the number of authors and then estimate whether and to what extent you provided more or less work than your co-authors. Only fill as many rows as you have papers. Do include papers where you were the sole author, your contribution for these will be 100%.

| Number of co-authors | Your contribution (percentage) | Year of publication  (2014-2016) |
| --- | --- | --- |
|  |  |  |
|  |  |  |
|  |  |  |
|  |  |  |
|  |  |  |
|  |  |  |
|  |  |  |
|  |  |  |
|  |  |  |
|  |  |  |
|  |  |  |
|  |  |  |
|  |  |  |
| **A total of 55 lines were available online** |  |  |
